# Alternative Splicing: A Key Mediator of Diabetic Vasculopathy

**DOI:** 10.3390/genes12091332

**Published:** 2021-08-27

**Authors:** Victoria A. Cornelius, Jenna R. Fulton, Andriana Margariti

**Affiliations:** The Wellcome-Wolfson Institute of Experimental Medicine, Belfast BT9 7BL, UK; vcornelius01@qub.ac.uk (V.A.C.); jfulton07@qub.ac.uk (J.R.F.)

**Keywords:** alternative splicing, cardiovascular disease, diabetic vasculopathy, atherosclerosis, isoforms, quaking, QKI, therapeutic strategies

## Abstract

Cardiovascular disease is the leading cause of death amongst diabetic individuals. Atherosclerosis is the prominent driver of diabetic vascular complications, which is triggered by the detrimental effects of hyperglycemia and oxidative stress on the vasculature. Research has extensively shown diabetes to result in the malfunction of the endothelium, the main component of blood vessels, causing severe vascular complications. The pathogenic mechanism in which diabetes induces vascular dysfunction, however, remains largely unclear. Alternative splicing of protein coding pre-mRNAs is an essential regulatory mechanism of gene expression and is accepted to be intertwined with cellular physiology. Recently, a role for alternative splicing has arisen within vascular health, with aberrant mis-splicing having a critical role in disease development, including in atherosclerosis. This review focuses on the current knowledge of alternative splicing and the roles of alternatively spliced isoforms within the vasculature, with a particular focus on disease states. Furthermore, we explore the recent elucidation of the alternatively spliced QKI gene within vascular cell physiology and the onset of diabetic vasculopathy. Potential therapeutic strategies to restore aberrant splicing are also discussed.

## 1. Introduction

Alternative splicing, the removal of introns followed by specific exon ligation prior to mRNA translation, allows for a single gene to coordinate the synthesis of a multitude of protein isoforms, all of which can vary in both their structural and functional characteristics. It is proposed that over 95% of human genes will undergo a form of alternative splicing [1]. Consequently, alternative splicing is an extensive source of diversity for both coding and non-coding RNAs; diversity that is greatly needed to achieve the higher degree of complexity, regarding gene expression, observed in humans. Furthermore, alternative splicing is a crucial regulatory mechanism of gene expression whether it be in a developmental, tissue-specific, or signal transduction manner. Aberrant alternative splicing, also known as mis-splicing, however, has been revealed to result in the disruption of normal cellular function and subsequently the onset of an array of human diseases including cardiovascular diseases, where the onset of diabetes has frequently been reported to be the result of abnormal transcripts [2,3]. Approaches to correct dysfunctional alternative splicing through targeting the pre-mRNA, the splicing factors, or the proteins themselves therefore hold great potential to become attractive therapeutic strategies to mitigate a range of disease phenotypes.

As we extensively discussed previously [4], endothelial cell dysfunction is a critical mediator of cardiovascular events, including diabetic cardiovascular complications. Cardiovascular diseases are consequently the leading cause of mortality amongst diabetic individuals, with several reports stating that cardiovascular disease death rates can reach up to 2–4 times higher amongst people with diabetes than without [5,6,7]. Typically, endothelial cells are vital to vascular health due to their roles in biological pathways such as blood vessel formation, regulating vascular tone and growth, preventing thrombosis, maintaining hemostatic balance, and regulating coagulant mechanisms [8,9]. Dysfunction, however, as exhibited in diabetes, results in endothelial cells with disturbed vascular tone, enhanced monocyte adhesion, delayed replication and excessive cell death, increased permeability, aberrant angiogenesis, reduced nitric oxide bioavailability and an increase in oxidative stress and reactive oxygen species [10,11,12,13,14]. Consequently, studies have shown endothelial cell dysfunction to be a prime instigator of atherosclerosis onset and progression. Atherosclerosis refers to the process of atherogenesis, the build-up of plaques on the inner lining of arterial walls [15]. These plaques, composed of substances found within the blood such as fat, cholesterol, and calcium, can harden overtime resulting in the narrowing of arteries and consequently the restriction of blood flow. Moreover, these plaques are also prone to rupture, subsequently triggering thrombosis. Consequently, atherosclerosis is known to result in vascular defects that contribute to ischemia, the central pathology of cardiovascular disease. Hyperglycemia is therefore a critical mediator of endothelial cell dysfunction and the subsequent associated vascular complications mediated through the onset of atherosclerosis. Furthermore, numerous studies have shown hyperglycemia to significantly alter the gene expression profiles of endothelial cells [16]. However, despite the widely accepted role of alternative splicing in regulating gene expression, little work has been done to explore the impact of alternative splicing on endothelial cells, including in the development of disease states.

Current approaches to prevent cardiovascular disease onset amongst individuals with diabetes largely focuses on the importance of glycaemic control. However, although glycaemic control can reduce the onset of complications, the correlation between diabetes and cardiovascular disease remains a significant health and economic burden globally [17], thus suggesting that well managed glycaemic control alone is not enough to solely prevent the onset of endothelial cell dysfunction and atherosclerosis development. Moreover, compared with non-diabetic individuals, diabetic cardiovascular disease patients are often less suitable for the conventional revascularization treatment approaches and consequently require more severe interventions, such as amputation or a transplant to manage vascular complications. Recently, data has been collected that infers glycaemic control to have worsened over the last decade across the United States, further emphasizing the necessity for a re-evaluation of current therapy approaches [18,19]. Due to the extensive involvement of endothelial cell dysfunction in cardiovascular disease onset amongst individuals with diabetes, the ability to repair and regenerate the endothelium may consequently offer a more effective therapeutic approach towards vascular complications for these individuals. Thus, research characterizing the role of alternative splicing in such endothelial cells could provide valuable insights allowing for the development of needed therapeutic strategies.

This review discusses the current knowledge of alternative splicing within endothelial cells with a particular focus on the role of alternative splicing in the pathogenesis of diabetic vasculopathy. Potential therapeutic avenues with promise to mitigate mis-splicing and restore the disease phenotype are also evaluated.

## 2. Alternative Splicing and the Vasculature

The human vasculature is a complex structure involving intricate interactions between various cell types, which are critical to maintain vascular homeostasis. Blood vessels are comprised of endothelial cells, which form the endothelium, vascular smooth muscle cells, and pericytes [20]. As mentioned earlier, the endothelium is the main component of the vasculature and has several obligatory roles, including the regulation of cell proliferation, vascular permeability, and vascular tone, however, it is also involved in numerous vascular processes, including angiogenesis [9,21,22]. Angiogenesis denotes the migration and proliferation of endothelial cells to form new blood vessels from a pre-existing vasculature, allowing for the supply of oxygen and nutrients. Furthermore, angiogenesis is crucial to vascular health due to having central roles in growth and development as well as wound healing [23]. As such, angiogenesis is vital for the maintenance of vascular homeostasis and its dysregulation has been implicated in the pathogenesis of several disorders, such as diabetic retinopathy, cancer, and rheumatoid arthritis. Angiogenesis has been extensively reviewed elsewhere previously; briefly, however, pro-angiogenic signalling, mediated by the release of growth factors such as vascular endothelial growth factor-A (VEGF-A), angiopoietin-2 (ANG-2) and transforming growth factor-β (TGF-β) [23,24], stimulates endothelial cell invasion into the extracellular matrix and guides the tip cell in forming the perfused tubule along with other factors. Neighboring cells of the tip cell, known as stalk cells, divide to elongate the stalk, establishing the lumen of the new vessel. Pericytes subsequently attach, resulting in a mature vessel [25]. Studies have shown angiogenesis to be regulated at both the transcriptional and post-transcriptional level. More recently, however, research has focused on the importance of alternatively spliced isoforms within this complex process, many of which are displayed in Table 1.

**Figure 1:**
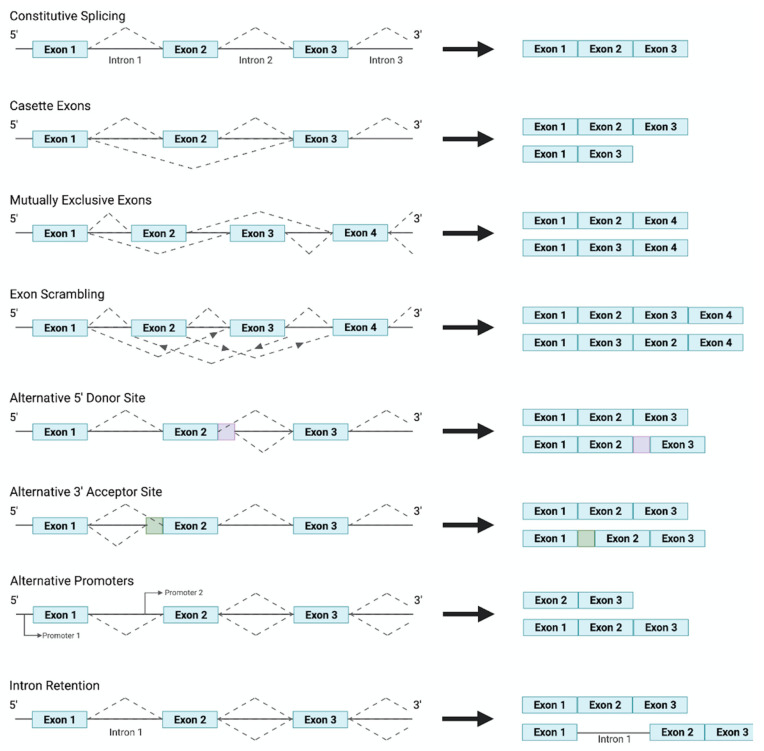
A schematic overview of the commonly observed splicing patterns responsible for the production of distinct transcripts from a singular gene.

Alternative splicing and the differing expression of isoforms are orchestrated through the induction of signal transduction pathways, by growth factors, which mediate the activation of specific splicing factors. Research has shown specific growth factors to regulate the action of splicing factor kinases, and thus splicing factors, through cell signaling molecules. Splicing factors, through interacting with the C terminal of RNA polymerase II, resulting in its recruitment to specific cis-acting splicing sequences, then coordinate the generation of different isoforms through the differential selection of 3′ Distal Splicing or 3′ Proximal Splicing sites. Moreover, several alternative splicing patterns have emerged, depicted in Figure 1, all of which can result in a plethora of protein isoforms with varying functions being generated from a single pre-mRNA transcript. The regulation and mechanisms of alternative splicing have been reviewed previously in [48].

Regarding vascular function, alternative splicing of VEGF-A, a vital factor of angiogenesis, is known to coordinate the synthesis of multiple distinct isoforms. The VEGF-A isoforms differ in their C terminal, contributing to their varying functions, and as such have been categorized into two distinct families. Members of the first, commonly denoted as VEGF-A_xxx_a, have been demonstrated to promote angiogenesis [2,3,49], with VEGF-A_165_ being the most abundant and important pro-angiogenic isoform derived from VEGF-A. Proximal splice-site selection in exon 8 is believed to co-ordinate the synthesis of pro-angiogenic VEGF-A_xxx_a isoforms, whilst distal splice-site selection results in the production of anti-angiogenic VEGF-A_xxx_b isoforms. Research has revealed growth factor TGF-β1 to stimulate p38 mitogen-activated protein kinase, activating the kinases CLK1 and CLK4 [50]. Both CLK1 and CLK4 were subsequently found to phosphorylate the splice factor SRP55, resulting in distal splice-site selection and consequently the generation of VEGF-A_xxx_b isoforms. Similarly, the same study showed SRP40 and ASF/SF2, two splice factors thought to be involved in determining the expression of the VEGF-A isoforms due to binding to the exonic splice enhancers in exon 8 [51], to also be phosphorylated by CLK1 and favor proximal splice-site selection. Unlike the VEGF-A_xxx_a isoforms, however, the members of the VEGF-A_xxx_b family are widely accepted to be inhibitors of angiogenesis and have been linked to numerous disease states including diabetic retinopathy and atherosclerosis [52].

VEGF-A signaling has been shown to promote macrophage infiltration and foam cell formation, both of which have central roles in the pathogenesis of atherosclerosis. Research investigating the link between VEGF-A splicing and atherosclerosis revealed a second isoform of VEGF-A_165_, referred to as VEGF-A_165_b, to be anti-angiogenic and heavily associated with the onset and development of atherosclerosis [53,54]. A recent study performed on apolipoprotein E deficient mice revealed that mice on a high fat diet exhibited a major shift in VEGF-A alternative splicing to VEGF-A_165_b, accredited to an upregulation of SRPK1 [53]. Furthermore, quantification of both aortic endothelial cells and macrophages revealed that the mice with an upregulation of VEGF-A_165_b exhibited characteristics associated with atherosclerosis pathogenesis [55]. Moreover, multiple studies have demonstrated the mis-splicing of VEGF-A resulting in an elevation of VEGF-A_165_b and a corresponding reduction in pro-angiogenic VEGF-A_165_ to contribute to ischemic cardiovascular disease. In addition, research has found the expression profile of VEGF-A spliced isoforms to vary amongst individuals with and without diabetes [56,57]. Furthermore, in a peripheral artery disease model, targeting upregulated VEGF-A_165_b reversed the impaired revascularization [58]. Similarly, studies have shown the potential of restoring altered VEGF-A splicing to protect blood vessels and ameliorate the associated diabetic vascular complications [59]. Restoring mis-splicing of VEGF-A therefore may have great therapeutic potential to restore vascular health. In particular, due to the association of aberrant VEGF-A splicing within atherosclerosis development, further research into VEGF-A splicing may reveal therapeutic strategies to target diabetic cardiovascular events.

Moreover, the aberrant function of alternative splice factor NOVA2, which typically has crucial roles in the regulation of endothelial cell organisation during angiogenesis [40], has shown to contribute to the vascular dysfunction observed in neurological diseases such as Alzheimer’s disease [60,61]. Alternative splicing of HNF1A has been isolated as a common cause of maturity-onset diabetes of the young (MODY), a disease that often presents hand in hand with endothelial cell dysfunction and microvascular complications, such as diabetic retinopathy, neuropathy, and nephropathy [62,63]. The generation of multiple transcripts from HNF1A is mainly caused by splicing of exons 2 and 4, however, variants have also been reported in the promoter, 5′UTR and 3′UTR [64]. Interestingly, a recent study exploring the consequences of HNF1A splicing in human endothelial cells derived from induced pluripotent stem cells found that compared with the control, the endothelial cells with aberrant HNF1A transcripts showed increased vascular permeability and expression of ICAM-1 in response to pro-inflammatory cytokine TNFα, contributing to endothelial dysfunction. Understanding the role of alternative splicing within the vasculature, as exampled below in the case of the QKI family, may therefore deepen our understanding of vascular health and vasculopathy, thus opening new avenues for disease treatment. This is especially important with regards to diseases such as diabetic endotheliopathy with current poor treatment strategies.

## 3. Alternative Splicing of QKI and Endothelial Cell Physiology

The QKI family is a prime example of isoforms originating from a single gene transcript but varying drastically in function. The QKI transcript encodes multiple RNA binding protein isoforms belonging to the Signal Transduction and Activation of RNA (STAR) family. All the transcribed QKI protein isoforms share a conserved KH RNA-binding motif domain, N-terminal Qua1 and C-terminal Qua2 domains, as well as multiple phosphorylation sites [65]. However, each generated QKI isoform differs in their carboxy-terminal end, resulting in their varying functions. Despite differing in their C terminal thirty amino acids, the three major isoforms produced, QKI-5, QKI-6, and QKI-7, have all shown to be implicated in vascular health. QKI was first suspected to be implicated in vasculature following the revelation that QKI null mice are embryonic lethal. The inability of QKI knockout mice embryos to develop past embryonic day 10.5 prompted further investigation, which revealed QKI silencing to trigger vascular defects through the development of immature endothelial tube structures and consequently abnormal vitelline vessels [66]. Subsequent analysis has since isolated the different alternatively spliced QKI isoforms to be expressed and crucial for vascular cell health [67].

The alternatively spliced isoform QKI-5, which largely resides in the nucleus, is believed to be the most prominently expressed QKI isoform within endothelial cells [68]. Research has shown QKI-5 to have roles in both differentiation and maintenance of typical physiology. In particular, the dysfunction observed in QKI null embryos was later found to be attributable to the loss of the QKI-5 splice site. Moreover, through STAT3 signaling, research has subsequently demonstrated QKI-5 to regulate CD144 stabilization and activate vascular endothelial growth factor receptor 2 (VEGFR2), thus acting as a key mediator of endothelial cell differentiation from induced pluripotent stem cells [69]. In addition, QKI-5 expression has been linked to appropriate endothelial cell function. Studies have revealed QKI-5 to bind to VE-cadherin and β-catenin [68], thus mediating and initiating their translation, both of which have extensive roles in maintaining endothelial cell-cell adhesion and barrier function. Furthermore, QKI-5 has been associated with neovascularization, blood flow recovery, and angiogenesis, further emphasizing the importance of the alternatively spliced QKI-5 isoform in vascular health. Similarly, research has confirmed that expression of both the QKI-5 and QKI-6 isoforms results in vascular cells that exhibit greater properties [70].

Recent work within our group, focused on diabetic vasculopathy, however, uncovered aberrant splicing of the QKI gene within endothelial cells to be responsible for and orchestrate diabetic endothelial cell dysfunction [71]. Witnessed through the onset of a disruption in the endothelial cell barrier, compromised angiogenesis and enhanced monocyte adhesion, we previously revealed the expression levels of two splicing regulators, namely CUG-BP and hnRNPM, to be drastically altered in response to glucose. Elevated levels of glucose, such as exhibited in individuals with diabetes, led to both the upregulation of CUG-BP and the downregulation of hnRNPM. The disruption of the CUG-BP and hnRNPM splicing factors under diabetic conditions was found to result in the significant upregulation of the alternatively spliced isoform QKI-7 through direct binding. Upregulation of QKI-7 was shown to result in the subsequent degradation of CD144, NLGN1 and TSG-6, three essential genes for vascular health. Investigation of QKI-7 knockdown in endothelial cells resulted in the restoration of the observed endothelial cell dysfunction as well as promoted reperfusion and blood flow recovery following hindlimb ischemia induced in diabetic mice. These findings therefore display aberrant splicing and the subsequent upregulation of QKI-7 to have a critical role in the pathogenic mechanism of diabetic vasculopathy. The ability of CUG-BP and hnRNPM to diametrically regulate QKI-7 in diabetes consequently offers a potential therapeutic avenue to restore vascular health in such individuals, whether through indirectly correcting the balance of the splicing factors or directly targeting QKI-7 expression for example.

## 4. Alternative Splicing Based Therapeutic Strategies

**Figure 2:**
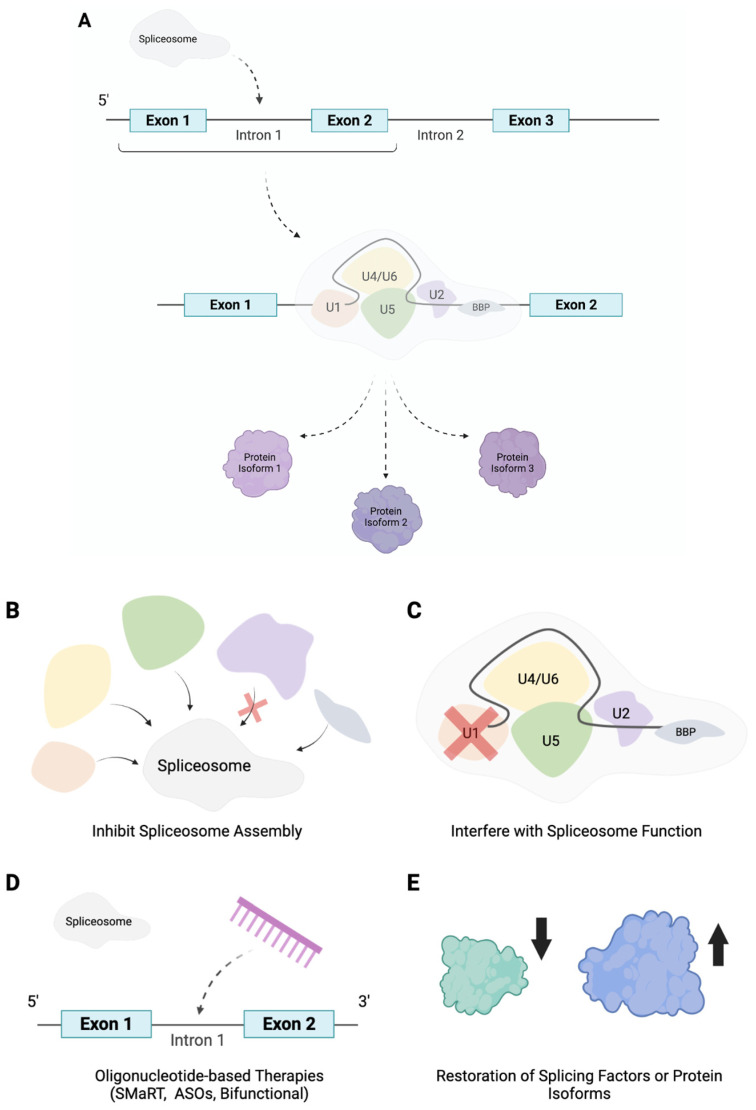
A schematic diagram simplifying the process of alternative splicing as well as depicting potential therapeutic strategies to mitigate mis-splicing. Briefly, the spliceosome, consisting of five crucial small nuclear ribonucleoproteins, as displayed in the figure, alongside other proteins catalyzes the splicing of pre-mRNA. Through coordinating the use of splice sites, the retention or exclusion of exons and any intron inclusion, alternative splicing results in the synthesis of multiple protein isoforms from one gene transcript (**A**). Aberrant alternative splicing has been shown to have a critical role in the pathogenesis of numerous diseases, consequently the design and development of therapeutics able to mitigate mis-splicing has emerged as a popular area of research. Two promising strategies that recently emerged revolve around the elucidation of compounds with the ability to obstruct the spliceosome. These include searching for drugs with the ability to inhibit spliceosome assembly or the interference of spliceosome function, as can be seen in (**B**,**C**). Moreover, the use of oligonucelotides as therapeutics, including spliceosome-mediated RNA trans-splicing (SMaRT) technologies, antisense oligonucleotides (ASOs) and bifunctional oligonucleotides, has attracted significant attention due to their ability to be designed to bind to a complementary sequence and trigger either the activation or inhibition of splicing events by sterically blocking or recruiting effectors to promote splicing (**D**). Similarly designing therapeutic strategies to restore the natural balance of splicing factors or generated spliced isoforms has shown great promise to reverse the disease phenotype and is therefore another encouraging therapeutic avenue to target mis-splicing (**E**).

Alternative splicing describes the directed combination of exons, mostly, from a pre-mRNA sequence resulting in the production of a manifold of different mRNAs from a singular gene as displayed in Figure 1. In general, alternative splicing is regulated through corresponding cis- and trans-acting regulatory sequences that together are responsible for either the inclusion or exclusion of the primary region. Moreover, intronic and exonic splicing enhancers coordinate the recruitment of splice factors, which organize the incorporation of a region within the mRNA. Intronic and exonic silencers, on the other hand, recruit splicing factors that facilitate transcript exclusion. Typically, these sites are located towards the 5′ and 3′ end of introns; however, an additional site, termed the branch point, can be found further upstream of the 3′ end of an intron. The spliceosome, containing the proteins and five small nuclear ribonucleoproteins required to catalyse splicing, is then ultimately responsible for either the use of alternative splice sites and the retention or exclusion of exons (as well as any intron inclusion) (Figure 2). As such a viable strategy to target mis-splicing would be to inhibit spliceosome assembly or interfere with spliceosome function. This notion has been heavily explored in the search for anti-cancer drugs. For example, research has isolated numerous compounds with the ability to target the spliceosome subunit SF3B1 protein [72,73,74], abolishing its conformation rearrangement and subsequently resulting in the correction of mis-splicing and alleviation of the disease phenotype.

Alternatively, mis-splicing could be corrected through interfering with specific splice sites thus redirecting and restoring the splice site selection. With this purpose in mind, oligonucleotide-based therapies are currently being investigated. Oligonucleotides are synthetic antisense molecules that bind to a complementary sequence, oligonucleotide-based therapies therefore involve the design of specific oligonucleotides capable of disrupting mis-splicing through binding to the transcript and either activating or inhibiting splicing events by sterically blocking or recruiting effectors to promote splicing. Inhibition of a splice site, for example, has great potential to modulate alternative splicing patterns and restore a range of disease phenotypes as it can be used to target splice sites created by a genetic mutation such as allowing for the restoration of a mRNA reading frame through coordinating the skipping of an exon that codes for a premature stop codon. Furthermore, exon skipping could also be used to coordinate the downregulation of a protein, thus allowing for the directed targeting of pathological isoforms. Due to their potential and thus popularity amongst researchers, a range of oligonucleotide-based therapies have been developed and extensively reviewed, including spliceosome-mediated RNA trans-splicing (SMaRT) technologies [75], antisense oligonucleotides (ASOs) [76] and bifunctional oligonucleotides [77] to name a few. Oligonucleotides therefore have great potential as therapeutics for a range of diseases through directly targeting specific splice sites.

Moreover, restoring the balance of splicing factors, through viral reintroduction for example, would provide an additional strategy to manage aberrant alternative splicing. Many pathologies, including vascular diseases, have been characterized by a downregulation of specific splicing factors. For example, as mentioned earlier, the downregulation of splicing factor hnRNPM in diabetic individuals contributes to the onset of endotheliopathy, its reintroduction would therefore be a viable solution to correct the subsequent mis-splicing of the QKI gene and alleviate the associated disease phenotype. Similarly blocking the action of specific generated spliced isoforms is another attractive method to alleviate associated dysfunction. Isoform specific RNA interference, as described in [78], could therefore also have great promise to alter ratios of alternatively spliced isoforms. In the same manner, targeting QKI-7 expression or function directly, as previously shown in [71], was also able to restore endothelial dysfunction and greatly recover reperfusion and blood flow following the introduction of hindlimb ischemia in diabetic mice.

Due to the high association of alternative splicing within disease states, it is undeniable that the correction of abnormal splicing holds great potential for future therapeutics. With the poor treatment strategies available for cardiovascular disease, including individuals with diabetic vasculopathy, this is therefore an exciting avenue of research. First, however, exploration of alternative splicing mechanisms in health and how its subsequent dysregulation results in disease states is needed to elucidate novel strategies. In the meantime, until the role of alternative splicing in cardiovascular disease has been fully evaluated, alternatively spliced variants could serve as important biomarkers. For example, an investigation earlier this year focused on stable and unstable coronary artery disease uncovered a significant difference in the expression of long and short RECK alternatively spliced variants [79]. Although further research is required to fully explore the pathogenic mechanism of RECK splicing in plaque vulnerability, the RECK splice variants in the meantime could nevertheless act as promising biomarkers to monitor atherosclerosis.

## 5. Conclusions

With the ever-increasing prevalence of diabetes, and the associated vascular dysfunction, the search for an effective treatment strategy is paramount. Due to the evident role of alternative splicing in controlling gene expression and therefore the manipulation of cellular phenotype, it is an attractive area of research to study physiological states. Recently studies have started to explore the role of alternative splicing within the vasculature and thus its role in distinguishing between health and disease. In particular, a clear association between diabetic vascular dysfunction and aberrant alternative splicing has emerged. Moreover, data indicates that the restoration of typical splicing in such scenarios can alleviate the linked vascular defects. This therefore highlights a new avenue of therapeutic strategies to combat a highly prevalent but poorly understood disease. Popular therapeutic strategies currently revolve around the counteraction of mis-splicing events through targeting the spliceosome, the use of complimentary oligonucleotide sequences, or the restoration of splicing factors or generated spliced isoforms. Data, however, has also emerged indicating that alternatively spliced isoforms could serve as effective biomarkers of disease states and progression. Nevertheless, research is still greatly needed to fully understand the role of alternative splicing within vascular health and simultaneously vasculopathies. This elucidation would not only allow for a greater comprehension of disease pathogenesis but also highlight avenues for the development of novel therapeutic approaches.

## Figures and Tables

**Table 1:** A table displaying the effect of alternative splicing on vascular health and function through the generation of differing spliced isoforms. Abbreviations: ECs, endothelial cells; ECM, extracellular matrix; NO, nitric oxide.

Gene	Alternatively Spliced Isoform	Function
***VEGF-A***	*VEGF-A_xxx_a*	
	*VEGF-A_111_*	Pro-angiogenic. [26,27], missing exons 6 and 7, readily diffusible [28]
	*VEGF-A_121_*	Pro-angiogenic. Regulates two different phosphorylating sites of VEGFR2, Tyr (1175) and Tyr (1214) [29], missing exons 6 and 7, readily diffusible [28]
	*VEGF-A_145_*	Pro-angiogenic. Binds to ECM and KDR/flk-1 receptor of ECs [30]
	*VEGF-A_165_*	Pro-angiogenic. Most abundant isoform and most potent initiator of angiogenesis. Activates receptor phosphorylation of VEGFR2 and NRP-1. Promotes the release of NO and prostacyclin [31], binds KDR/flk-1 with VEGF-A_145_ [30]
	*VEGF-A_183_*	Pro-angiogenic. Least abundant [32]
	*VEGF-A_189_*	Pro-angiogenic. Linked to tumorigenesis [33]
	*VEGF-A_206_*	Pro-angiogenic. Strongly binds to ECM [34]
	*VEGF-A_xxx_b*	
	*VEGF-A_121_b*	Anti-angiogenic. Inhibits migration of ECs. Reduces xenografted tumor growth [35]
	*VEGF-A_145_b*	Anti-angiogenic
	*VEGF-A_165_b*	Anti-angiogenic. Neuroprotective and cytoprotective properties [36,37]. Similar binding affinity of VEGF-A_165_a but does not activate phosphorylation of VEGFR2 and NRP-1 [38]
	*VEGF-A_189_b*	Anti-angiogenic
**HNF1A**		Transcriptional activator that regulates insulin production and stimulates the transcription of other liver-specific genes CYP1A2, CYP2E1 and CYP3A11 [39]
**NOVA2**		Pro-angiogenic. Controls the organization of the endothelial lumen. Is also present in neural cells and important for the development of the nervous system [40]
**Fibronectin**	EDA-FN, EDB-FN	Pro-angiogenic. Component of ECM, involved in vascular remodelling, and inhibits oxidative stress [41]
***NRPS***	NRP-1	
	*_s11_NRP1, _s12_NRP1, _sIII_NRP1*	Anti-angiogenic. Lacks transmembrane domain and cytoplasmic tail [42]
	*& _sIV_NRP1*	
	*NRP1-∆7*	Anti-angiogenic. Deletion of seven amino acids in exon 11. Impairs glycosylation of NRP-1 [43]
	*NRP1-∆E4 & NRP1-∆E5*	Anti-angiogenic. Altered glycosylation and endocytic movement [44]
	NRP-2	
	*_s9_NRP2*	Results from intron 9 retention. Inhibits VEGF-C/NRP2 oncogenic signaling [45]
**Vasohibins**	Vasohibin-1	
	*VASH1A*	Anti-angiogenic. Linked to tumorigenesis [46]
	*VASHA1B*	Anti-angiogenic. Lacks exons 6–8. Involved in heparin binding [46]
	Vasohibin-2	
	*290aa & 355aa*	Anti-angiogenic. Full function unclear [47]

## References

[1] Jiang W., Chen L. (2020). Alternative splicing: Human disease and quantitative analysis from high-throughput sequencing. Comput. Struct. Biotechnol. J..

[2] Dlamini Z., Mokoena F., Hull R. (2017). Abnormalities in alternative splicing in diabetes: Therapeutic targets. J. Mol. Endocrinol..

[3] Mironidou-Tzouveleki M., Tsartsalis S., Tomos C. (2011). Vascular endothelial growth factor (VEGF) in the pathogenesis of diabetic nephropathy of type 1 diabetes mellitus. Curr. Drug Targets.

[4] Cornelius V.A., Yacoub A., Kelaini S., Margariti A. (2021). Diabetic endotheliopathy: RNA-binding proteins as new therapeutic targets. Int. J. Biochem. Cell Biol..

[5] Leon B.M., Maddox T.M. (2015). Diabetes and cardiovascular disease: Epidemiology, biological mechanisms, treatment recommendations and future research. World J. Diabetes.

[6] American Heart Association (2021). Cardiovascular Disease and Diabetes. https://www.heart.org/en/health-topics/diabetes/diabetes-complications-and-risks/cardiovascular-disease--diabetes.

[7] Rivellese A.A., Riccardi G., Vaccaro O. (2010). Cardiovascular risk in women with diabetes. Nutr. Metab. Cardiovasc. Dis..

[8] Davignon J., Ganz P. (2004). Role of endothelial dysfunction in atherosclerosis. Circulation.

[9] Sena C.M., Pereira A.M., Seiça R. (2013). Endothelial dysfunction—A major mediator of diabetic vascular disease. Biochim. Biophys. Acta (BBA)-Mol. Basis Dis..

[10] Duffy A., Liew A., O’Sullivan J., Avalos G., Samali A., O’Brien T. (2006). Distinct Effects of High-Glucose Conditions on Endothelial Cells of Macrovascular and Microvascular Origins. Endothelium.

[11] Hou C.J.-Y., Tsai C.-H., Su C.-H., Wu Y.-J., Chen S.-J., Chiu J.-J., Shiao M.-S., Yeh H.-I. (2008). Diabetes Reduces Aortic Endothelial Gap Junctions in ApoE-deficient Mice: Simvastatin Exacerbates the Reduction. J. Histochem. Cytochem..

[12] Brouwers O., Niessen P.M., Haenen G., Miyata T., Brownlee M., Stehouwer C.D., De Mey J.G., Schalkwijk C.G. (2010). Hyperglycaemia-induced impairment of endothelium-dependent vasorelaxation in rat mesenteric arteries is mediated by intracellular methylglyoxal levels in a pathway dependent on oxidative stress. Diabetologia.

[13] Karbach S., Jansen T., Horke S., Heeren T., Scholz A., Coldewey M., Karpi A., Hausding M., Kröller-Schön S., Oelze M. (2012). Hyperglycemia and oxidative stress in cultured endothelial cells—A comparison of primary endothelial cells with an immortalized endothelial cell line. J. Diabetes Its Complicat..

[14] Kemeny S.F., Figueroa D.S., Clyne A.M. (2013). Hypo- and hyperglycemia impair endothelial cell actin alignment and nitric oxide synthase activation in response to shear stress. PLoS ONE.

[15] Rafieian-Kopaei M., Setorki M., Doudi M., Baradaran A., Nasri H. (2014). Atherosclerosis: Process, indicators, risk factors and new hopes. Int. J. Prev. Med..

[16] Moradipoor S., Ismail P., Etemad A., Wan Sulaiman W.A., Ahmadloo S. (2016). Expression Profiling of Genes Related to Endothelial Cells Biology in Patients with Type 2 Diabetes and Patients with Prediabetes. Biomed. Res. Int..

[17] Foundation, B.H. (2021). Facts and Figures. https://www.bhf.org.uk/what-we-do/news-from-the-bhf/contact-the-press-office/facts-and-figures.

[18] Chiasson J.-L., Josse R.G., Gomis R., Hanefeld M., Karasik A., Laakso M., STOP-NIDDM Trial Research Group (2003). Acarbose Treatment and the Risk of Cardiovascular Disease and Hypertension in Patients With Impaired Glucose ToleranceThe STOP-NIDDM Trial. JAMA.

[19] Hanefeld M., Cagatay M., Petrowitsch T., Neuser D., Petzinna D., Rupp M. (2004). Acarbose reduces the risk for myocardial infarction in type 2 diabetic patients: Meta-analysis of seven long-term studies. Eur. Heart J..

[20] Bergers G., Song S. (2005). The role of pericytes in blood-vessel formation and maintenance. Neuro Oncol..

[21] Shi Y., Vanhoutte P.M. (2017). Macro- and microvascular endothelial dysfunction in diabetes. J. Diabetes.

[22] Eelen G., de Zeeuw P., Simons M., Carmeliet P. (2015). Endothelial cell metabolism in normal and diseased vasculature. Circ. Res..

[23] Failla C.M., Carbo M., Morea V. (2018). Positive and Negative Regulation of Angiogenesis by Soluble Vascular Endothelial Growth Factor Receptor-1. Int. J. Mol. Sci..

[24] Xin H., Zhong C., Nudleman E., Ferrara N. (2016). Evidence for Pro-angiogenic Functions of VEGF-Ax. Cell.

[25] Hagedorn M., Balke M., Schmidt A., Bloch W., Kurz H., Javerzat S., Rousseau B., Wilting J., Bikfalvi A. (2004). VEGF coordinates interaction of pericytes and endothelial cells during vasculogenesis and experimental angiogenesis. Dev. Dyn..

[26] Bowler E., Oltean S. (2019). Alternative Splicing in Angiogenesis. Int. J. Mol. Sci..

[27] Mamer S.B., Wittenkeller A., Imoukhuede P.I. (2020). VEGF-A splice variants bind VEGFRs with differential affinities. Sci. Rep..

[28] Krilleke D., DeErkenez A., Schubert W., Giri I., Robinson G.S., Ng Y.S., Shima D.T. (2007). Molecular mapping and functional characterization of the VEGF164 heparin-binding domain. J. Biol. Chem..

[29] Shiying W., Boyun S., Jianye Y., Wanjun Z., Ping T., Jiang L., Hongyi H. (2017). The Different Effects of VEGFA121 and VEGFA165 on Regulating Angiogenesis Depend on Phosphorylation Sites of VEGFR2. Inflamm. Bowel. Dis..

[30] Poltorak Z., Cohen T., Sivan R., Kandelis Y., Spira G., Vlodavsky I., Keshet E., Neufeld G. (1997). VEGF145, a secreted vascular endothelial growth factor isoform that binds to extracellular matrix. J. Biol. Chem..

[31] Neagoe P.E., Lemieux C., Sirois M.G. (2005). Vascular endothelial growth factor (VEGF)-A165-induced prostacyclin synthesis requires the activation of VEGF receptor-1 and -2 heterodimer. J. Biol. Chem..

[32] Neufeld G., Cohen T., Gengrinovitch S., Poltorak Z. (1999). Vascular endothelial growth factor (VEGF) and its receptors. FASEB J..

[33] Nakamura M., Abe Y., Tokunaga T. (2002). Pathological significance of vascular endothelial growth factor A isoform expression in human cancer. Pathol. Int..

[34] Hoar F.J., Lip G.Y., Belgore F., Stonelake P.S. (2004). Circulating levels of VEGF-A, VEGF-D and soluble VEGF-A receptor (sFIt-1) in human breast cancer. Int. J. Biol. Markers.

[35] Eymin B., Boudria A., Abou-Faycal C., Feige J.-J., Pagès G., Soncin F. (2014). VEGF-A Splice Variants: Do They Play a Role in Tumor Responses to Anti-angiogenic Therapies?. Molecular Mechanisms of Angiogenesis: From Ontogenesis to Oncogenesis.

[36] Beazley-Long N., Hua J., Jehle T., Hulse R.P., Dersch R., Lehrling C., Bevan H., Qiu Y., Lagrèze W.A., Wynick D. (2013). VEGF-A165b is an endogenous neuroprotective splice isoform of vascular endothelial growth factor A in vivo and in vitro. Am. J. Pathol..

[37] Magnussen A.L., Rennel E.S., Hua J., Bevan H.S., Beazley Long N., Lehrling C., Gammons M., Floege J., Harper S.J., Agostini H.T. (2010). VEGF-A165b is cytoprotective and antiangiogenic in the retina. Invest. Ophthalmol. Vis. Sci..

[38] Ngo D.T.M., Farb M.G., Kikuchi R., Karki S., Tiwari S., Bigornia S.J., Bates D.O., LaValley M.P., Hamburg N.M., Vita J.A. (2014). Antiangiogenic Actions of Vascular Endothelial Growth Factor-A165b, an Inhibitory Isoform of Vascular Endothelial Growth Factor-A, in Human Obesity. Circulation.

[39] Chi Y.-I., Frantz J.D., Oh B.-C., Hansen L., Dhe-Paganon S., Shoelson S.E. (2002). Diabetes Mutations Delineate an Atypical POU Domain in HNF-1α. Mol. Cell.

[40] Giampietro C., Deflorian G., Gallo S., Di Matteo A., Pradella D., Bonomi S., Belloni E., Nyqvist D., Quaranta V., Confalonieri S. (2015). The alternative splicing factor Nova2 regulates vascular development and lumen formation. Nat. Commun..

[41] Gortan Cappellari G., Barazzoni R., Cattin L., Muro A.F., Zanetti M. (2016). Lack of Fibronectin Extra Domain A Alternative Splicing Exacerbates Endothelial Dysfunction in Diabetes. Sci. Rep..

[42] Cackowski F.C., Xu L., Hu B., Cheng S.Y. (2004). Identification of two novel alternatively spliced Neuropilin-1 isoforms. Genomics.

[43] Hendricks C., Dubail J., Brohée L., Delforge Y., Colige A., Deroanne C. (2016). A Novel Physiological Glycosaminoglycan-Deficient Splice Variant of Neuropilin-1 Is Anti-Tumorigenic In Vitro and In Vivo. PLoS ONE.

[44] Huang X., Ye Q., Chen M., Li A., Mi W., Fang Y., Zaytseva Y.Y., O’Connor K.L., Vander Kooi C.W., Liu S. (2019). N-glycosylation-defective splice variants of neuropilin-1 promote metastasis by activating endosomal signals. Nat. Commun..

[45] Parker M.W., Linkugel A.D., Goel H.L., Wu T., Mercurio A.M., Vander Kooi C.W. (2015). Structural basis for VEGF-C binding to neuropilin-2 and sequestration by a soluble splice form. Structure.

[46] Horie S., Suzuki Y., Kobayashi M., Kadonosono T., Kondoh S., Kodama T., Sato Y. (2016). Distinctive role of vasohibin-1A and its splicing variant vasohibin-1B in tumor angiogenesis. Cancer Gene Ther..

[47] Sato Y., Sonoda H. (2007). The vasohibin family: A negative regulatory system of angiogenesis genetically programmed in endothelial cells. Arter. Thromb. Vasc. Biol..

[48] Chen M., Manley J.L. (2009). Mechanisms of alternative splicing regulation: Insights from molecular and genomics approaches. Nat. Rev. Mol. Cell Biol..

[49] Lambrechts D., Storkebaum E., Morimoto M., Del-Favero J., Desmet F., Marklund S.L., Wyns S., Thijs V., Andersson J., van Marion I. (2003). VEGF is a modifier of amyotrophic lateral sclerosis in mice and humans and protects motoneurons against ischemic death. Nat. Genet..

[50] Nowak D.G., Woolard J., Amin E.M., Konopatskaya O., Saleem M.A., Churchill A.J., Ladomery M.R., Harper S.J., Bates D.O. (2008). Expression of pro- and anti-angiogenic isoforms of VEGF is differentially regulated by splicing and growth factors. J. Cell Sci..

[51] Carter J.G., Cherry J., Williams K., Turner S., Bates D.O., Churchill A.J. (2011). Splicing factor polymorphisms, the control of VEGF isoforms and association with angiogenic eye disease. Curr. Eye Res..

[52] Ye X., Abou-Rayyah Y., Bischoff J., Ritchie A., Sebire N.J., Watts P., Churchill A.J., Bates D.O. (2016). Altered ratios of pro- and anti-angiogenic VEGF-A variants and pericyte expression of DLL4 disrupt vascular maturation in infantile haemangioma. J. Pathol..

[53] Zhao N., Zhang J. (2018). Role of alternative splicing of VEGF-A in the development of atherosclerosis. Aging.

[54] Camaré C., Pucelle M., Nègre-Salvayre A., Salvayre R. (2017). Angiogenesis in the atherosclerotic plaque. Redox Biol..

[55] Insull W. (2009). The Pathology of Atherosclerosis: Plaque Development and Plaque Responses to Medical Treatment. Am. J. Med..

[56] Zafar M.I., Mills K., Ye X., Blakely B., Min J., Kong W., Zhang N., Gou L., Regmi A., Hu S.Q. (2018). Association between the expression of vascular endothelial growth factors and metabolic syndrome or its components: A systematic review and meta-analysis. Diabetol. Metab. Syndr..

[57] Giannarelli C., Alique M., Rodriguez D.T., Yang D.K., Jeong D., Calcagno C., Hutter R., Millon A., Kovacic J.C., Weber T. (2014). Alternatively spliced tissue factor promotes plaque angiogenesis through the activation of hypoxia-inducible factor-1α and vascular endothelial growth factor signaling. Circulation.

[58] Kikuchi R., Nakamura K., MacLauchlan S., Ngo D.T.-M., Shimizu I., Fuster J.J., Katanasaka Y., Yoshida S., Qiu Y., Yamaguchi T.P. (2014). An antiangiogenic isoform of VEGF-A contributes to impaired vascularization in peripheral artery disease. Nat. Med..

[59] Oltean S., Qiu Y., Ferguson J.K., Stevens M., Neal C., Russell A., Kaura A., Arkill K.P., Harris K., Symonds C. (2015). Vascular Endothelial Growth Factor-A_165_b Is Protective and Restores Endothelial Glycocalyx in Diabetic Nephropathy. J. Am. Soc. Nephrol..

[60] Zacchigna S., Lambrechts D., Carmeliet P. (2008). Neurovascular signalling defects in neurodegeneration. Nat. Rev. Neurosci..

[61] Joutel A., Corpechot C., Ducros A., Vahedi K., Chabriat H., Mouton P., Alamowitch S., Domenga V., Cécillion M., Maréchal E. (1996). Notch3 mutations in CADASIL, a hereditary adult-onset condition causing stroke and dementia. Nature.

[62] Kachamakova-Trojanowska N., Stepniewski J., Dulak J. (2019). Human iPSCs-Derived Endothelial Cells with Mutation in HNF1A as a Model of Maturity-Onset Diabetes of the Young. Cells.

[63] Balamurugan K., Bjørkhaug L., Mahajan S., Kanthimathi S., Njølstad P.R., Srinivasan N., Mohan V., Radha V. (2016). Structure-function studies of HNF1A (MODY3) gene mutations in South Indian patients with monogenic diabetes. Clin. Genet..

[64] Ellard S., Colclough K. (2006). Mutations in the genes encoding the transcription factors hepatocyte nuclear factor 1 alpha (HNF1A) and 4 alpha (HNF4A) in maturity-onset diabetes of the young. Hum. Mutat..

[65] Teplova M., Hafner M., Teplov D., Essig K., Tuschl T., Patel D.J. (2013). Structure-function studies of STAR family Quaking proteins bound to their in vivo RNA target sites. Genes Dev..

[66] Noveroske J.K., Lai L., Gaussin V., Northrop J.L., Nakamura H., Hirschi K.K., Justice M.J. (2002). Quaking is essential for blood vessel development. Genesis.

[67] van Mil A., Grundmann S., Goumans M.-J., Lei Z., Oerlemans M.I., Jaksani S., Doevendans P.A., Sluijter J.P.G. (2012). MicroRNA-214 inhibits angiogenesis by targeting Quaking and reducing angiogenic growth factor release. Cardiovasc. Res..

[68] de Bruin R.G., van der Veer E.P., Prins J., Lee D.H., Dane M.J., Zhang H., Roeten M.K., Bijkerk R., de Boer H.C., Rabelink T.J. (2016). The RNA-binding protein quaking maintains endothelial barrier function and affects VE-cadherin and beta-catenin protein expression. Sci. Rep..

[69] Cochrane A., Kelaini S., Tsifaki M., Bojdo J., Vila-Gonzalez M., Drehmer D., Caines R., Magee C., Eleftheriadou M., Hu Y. (2017). Quaking Is a Key Regulator of Endothelial Cell Differentiation, Neovascularization, and Angiogenesis. Stem. Cells.

[70] Caines R., Cochrane A., Kelaini S., Vila-Gonzalez M., Yang C., Eleftheriadou M., Moez A., Stitt A.W., Zeng L., Grieve D.J. (2019). The RNA-binding protein QKI controls alternative splicing in vascular cells, producing an effective model for therapy. J. Cell Sci..

[71] Yang C., Eleftheriadou M., Kelaini S., Morrison T., González M.V., Caines R., Edwards N., Yacoub A., Edgar K., Moez A. (2020). Targeting QKI-7 in vivo restores endothelial cell function in diabetes. Nat. Commun..

[72] Shi Y., Bray W., Smith A.J., Zhou W., Calaoagan J., Lagisetti C., Sambucetti L., Crews P., Lokey R.S., Webb T.R. (2020). An exon skipping screen identifies antitumor drugs that are potent modulators of pre-mRNA splicing, suggesting new therapeutic applications. PLoS ONE.

[73] Kaida D., Motoyoshi H., Tashiro E., Nojima T., Hagiwara M., Ishigami K., Watanabe H., Kitahara T., Yoshida T., Nakajima H. (2007). Spliceostatin A targets SF3b and inhibits both splicing and nuclear retention of pre-mRNA. Nat. Chem. Biol..

[74] Kotake Y., Sagane K., Owa T., Mimori-Kiyosue Y., Shimizu H., Uesugi M., Ishihama Y., Iwata M., Mizui Y. (2007). Splicing factor SF3b as a target of the antitumor natural product pladienolide. Nat. Chem. Biol..

[75] Berger A., Maire S., Gaillard M.C., Sahel J.A., Hantraye P., Bemelmans A.P. (2016). mRNA trans-splicing in gene therapy for genetic diseases. Wiley Interdiscip. Rev. RNA.

[76] Havens M.A., Hastings M.L. (2016). Splice-switching antisense oligonucleotides as therapeutic drugs. Nucleic Acids Res..

[77] Bhattarai U., Hsieh W.-C., Yan H., Guo Z.-F., Shaikh A.Y., Soltani A., Song Y., Ly D.H., Liang F.-S. (2020). Bifunctional small molecule-oligonucleotide hybrid as microRNA inhibitor. Bioorganic Med. Chem..

[78] Fuchs A., Riegler S., Ayatollahi Z., Cavallari N., Giono L.E., Nimeth B.A., Mutanwad K.V., Schweighofer A., Lucyshyn D., Barta A. (2021). Targeting alternative splicing by RNAi: From the differential impact on splice variants to triggering artificial pre-mRNA splicing. Nucleic Acids Res..

[79] Vancheri C., Morini E., Prandi F.R., Alkhoury E., Celotto R., Romeo F., Novelli G., Amati F. (2021). Two RECK Splice Variants (Long and Short) Are Differentially Expressed in Patients with Stable and Unstable Coronary Artery Disease: A Pilot Study. Genes.

